# Inhibition of SARS-CoV-2 Spike Protein Pseudotyped Virus Infection Using ACE2-Tethered Micro/Nanoparticles

**DOI:** 10.3390/bioengineering10060652

**Published:** 2023-05-26

**Authors:** Soha Y. Alkhaldi, Ian Peng, Ching-An Peng

**Affiliations:** Department of Chemical and Biological Engineering, University of Idaho, Moscow, ID 83844, USA

**Keywords:** SARS-CoV-2, ACE2, spike protein, pseudotyped lentivirus, functionalized particles, core streptavidin, HEK293T

## Abstract

Coronavirus disease 2019 (COVID-19) has caused a global pandemic of severe acute respiratory syndrome coronavirus 2 (SARS-CoV-2). The viral infection is reliant upon the binding between angiotensin-converting enzyme 2 receptor (ACE2) and spike protein (S). Therefore, ACE2 is a key receptor for SARS-CoV-2 to infect the host. Nonetheless, as SARS-CoV-2 is constantly mutating into new variants that cause high infection rates, the development of prophylactic and therapeutic approaches remains a necessity to continue fighting mutated SARS-CoV-2 variants. In this study, ACE2-streptavidin fusion proteins expressed by recombinant DNA technology were anchored on biotinylated fluorescent polystyrene particles of various sizes ranging from 0.15 to 5 µm. The ACE2-tethered micro/nanoparticles were shown to prevent spike protein pseudotyped lentivirus entry into ACE2-expressing HEK293T cells. Compared to ACE2 in soluble form, micro-sized particles (2 and 5 µm) immobilized with ACE2 interfered more efficiently with viral attachment, entry, and the ensuing infection. Our results showed that particles functionalized with ACE2 could be used as efficient decoys to block the infection of SARS-CoV-2 strains.

## 1. Introduction

The outbreak of emerging infectious diseases is an undeniable threat to human health. The severe acute respiratory syndrome coronavirus (SARS-CoV), for instance, first emerged and gave rise to a major pandemic in 2003 [1]. In December 2019, severe acute respiratory syndrome coronavirus 2 (SARS-CoV-2) arose again and caused coronavirus disease 2019 (COVID-19) outbreaks. Because this virus notoriously spreads rapidly from person to person and causes illnesses like pneumonia and numerous complications such as organ failures, it has resulted in high mortality rates [2]. By now, we know that SARS-CoV-2 enters the host cell through the angiotensin-converting enzyme 2 (ACE2) receptor [3], which is a protein that expresses on the surface of human respiratory epithelial cells [4]. The interaction with ACE2 is the major key mechanism that facilitates the early transmission of SARS-CoV-2 to the host [5], resulting in high viral infectivity [6]. The COVID-19 vaccine development has achieved tremendous success, but the development of mutant SARS-CoV-2 strains combined with the rather slow vaccination process globally has made the world vulnerable to COVID-19 threats [7,8,9,10]. According to the CDC, fully vaccinated populations in the United States reached 69% in March 2023 [11]. Since the risk of emerging new variants of SARS-CoV-2 and the possibility of future coronavirus outbreaks remain, it is necessary to continue developing prophylactic and therapeutic approaches to protect both unvaccinated and vaccinated individuals. Many researchers have focused on the role of ACE2 in the SARS-CoV-2 infection. One of the promising strategies to neutralize viral infection is based on the administration of recombinant ACE2 to inhibit virus-cell binding competitively via the spike protein [12]. Clinical-grade soluble ACE2 has been used to block SARS-CoV-2 entry into Vero cells in a dose-dependent manner, but very high concentrations of soluble ACE2 (up to 200 µg/mL) were required to inhibit SARS-CoV-2 infection [13,14]. Human recombinant soluble ACE2 protein bound to Ig-Fc has also been used to prevent SARS-CoV-2 infection, but recent studies have shown that soluble ACE2 has a short half-life, thereby limiting their effectiveness [15,16,17]. Furthermore, increased ACE2 concentrations may increase vulnerability to SARS-CoV-2 infection, particularly in patients with cardiac disease, hypertension, or diabetes who are taking ACE2-increasing drugs [18,19,20,21].

Nanotechnology has been used in medicine to prevent, diagnose, and treat diseases. The emergence of viral diseases has encouraged many scientists to utilize nanotechnology tools as potential therapeutics for viruses [22]. Porous gold nanoparticles, for example, have been used to inactivate the influenza A virus [23], and silica nanoparticles have been used as a vaccine carrier for a genetically engineered HIV-1 envelope trimer [24]. The effective delivery of particles via the pulmonary route is dependent on the size of the particles. It was reported that porous particles larger than 3 μm delivered to the lungs could escape from phagocytic clearance [25]. Additionally, the particle with a size between 1 and 5 µm can deposit in the alveoli [26], which is an optimal drug target site because it is the natural route of SARS-CoV-2 transmission. Nevertheless, with the knowledge that proteins immobilized on the surface of nanoparticles can increase protein activity, stability, and functionality rather than soluble proteins [27,28,29], ACE2 immobilized on micro/nanoparticles could be efficient decoys to eliminate SARS-CoV-2 infection and could have an extended half-life in comparison to soluble ACE2. In this study, ACE2 and core streptavidin (coreSA) gene sequences were cloned in a pET-30a(+) plasmid, and the ACE2-coreSA fusion protein was immobilized on the surface of biotinylated particles via biotin-streptavidin binding. For biosafety level-2 operation, spike protein (*S*) pseudotyped virions with a lentiviral core and a green fluorescent protein reporter were produced using plasmid co-transfection technology. Our results demonstrated that the entry of *S*-pseudotyped lentivirus to ACE2-expressing HEK293T cells was significantly blocked by 2 and 5-µm polystyrene particles bound with ACE2 in comparison with the case using soluble ACE2 alone.

## 2. Materials and Methods

### 2.1. Construction of Human ACE2-Core Streptavidin Encoding Plasmid (ACE2-coreSA)

**Figure 1:**
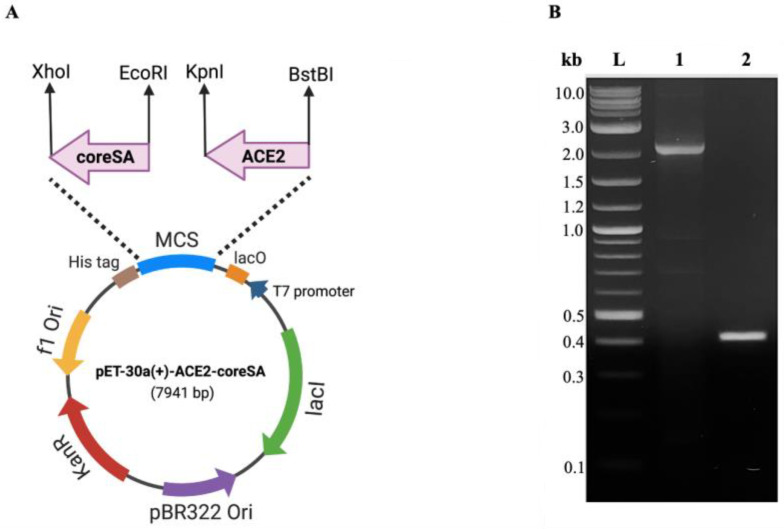
(**A**) The circular map shows the multiple cloning sites of pET-30a(+) for the ACE2-coreSA fusion gene. CoreSA was inserted between XhoI and EcoRI; human ACE2 was inserted between BstBI and KpnI; (**B**) DNA gel electrophoresis of human ACE2 (~2.1 kb) (lane 1), coreSA (~0.4 kb) (lane 2), and DNA ladder (L).

Using the polymerase chain reaction technique (PCR), the cDNA of angiotensin-converting enzyme 2 (ACE2) was cloned from pcDNA3.1-hACE2 (Addgene, Watertown, MA, USA) with forward primer (5′-ATTAATTCGAAACTGCTGCTCAGTCCACC-3′), and reverse primer (5′-ATTAAGGTACCGGAAACAGGGGGCTGGTT-3′) (Integrated DNA Technologies, Coralville, IA, USA). High-fidelity Phusion DNA polymerase (New England BioLabs, Ipswich, MA, USA) was used in the reaction, which was carried out using a thermocycler (T-100, Bio-Rad, Hercules, CA, USA) after an initial denaturation at 98 °C for one minute, followed by 34 cycles of denaturation at 98 °C for 15 s, annealing at 59 °C for 2 min, extension for 30 s at 72 °C, and final extension for 5 min at 72 °C. The cDNA of core streptavidin (coreSA) was cloned out from pSTE2-215 (yoI) plasmid [30] with forward primer (5′-AGATCCGAATTCGGTGCT GCTGAAGCAGG-3′) and reverse primer (5′-ATTATACTCGAGGGAGGCGGCGGACGGCT-3′) (Integrated DNA Technologies), with an initial denaturation at 98 °C for 10 s, followed by 34 cycles of denaturation at 98 °C for 30 s, annealing at 62 °C for 30 s, and extension at 72 °C for 15 s, and final extension at 72 °C for 5 min. For PCR confirmation, PCR products were purified by using the Monarch PCR and DNA cleanup kit (New England BioLabs) and run on 1% agarose (Thermo Fisher Scientific, Waltham, MA, USA) gel for electrophoresis. The restriction enzymes XhoI and EcoRI (New England BioLabs) were used to cut the insert (coreSA) and then ligated into pET-30a(+) (Addgene) by T4 DNA ligase (New England BioLabs) to get the desired plasmid (pET-30a-coreSA). To create pET-30a-ACE2 plasmid, the ACE2 PCR product was digested with BstBI and KpnI (New England BioLabs) and ligated into the pET-30a-vector. In order to create a pET30a-ACE2-coreSA plasmid, the ACE2 PCR product was digested with BstBI and KpnI and ligated into the pET-30a-coreSA vector. The competent *E. coli* 5α (New England BioLabs) was used to amplify the final ligation product by transformation. Then, the Plasmid Miniprep kit (QIAGEN, Germantown, MD, USA) was used for purification. As shown in Figure 1A, the coreSA and ACE2 gene sequences were inserted into the vector pET-30a(+) to create the ACE2-coreSA encoding recombinant plasmid (pET-30a-ACE2-coreSA).

### 2.2. Expression of ACE2-coreSA Fusion Protein

According to the previously reported method [31], the competent *E. coli* Lemo21(DE3) (New England BioLabs) was used for the transformation and protein expression of the constructed pET-30a-ACE2-coreSA vector. After transformation, the bacterial cells were spread on an agar plate containing 50 mg/L of kanamycin (Santa Cruz Biotechnology, Dallas, TX, USA) and cultured for an overnight period at 37 °C. For the creation of starting cultures, one colony was selected from the agar plate, resuspended in 5 mL of lysogeny broth (LB) media (Sigma-Aldrich, St. Louis, MO, USA), treated with 50 mg/L of kanamycin, and allowed to develop overnight at 37 °C. The 0.5 mL of starter culture was diluted to make 1 liter of LB media, which was then fortified with 0.5 mM of L-rhamnose (New England BioLabs) and 50 mg/L of kanamycin. After the optical density reading at 600 nm (OD_600_) reached 0.6, 0.4 mM isopropyl-β-D-thiogalactopyranoside (IPTG, Sigma-Aldrich) was added to the cell culture, followed by an overnight shake at 22 °C with 225 rpm. Bacterial cells were centrifuged at 4600× *g* for 14 min. The cell pellet was lysed with nonionic detergent B-PER (Thermo Fisher Scientific) with protease inhibitor EDTA-free (Thermo Fisher Scientific), and 50 mM Tris-HCl (pH 7.4) (Promega, Madison, WI, USA), which was then incubated at room temperature for 15 min. Then, the bacterial lysate was sonicated on ice for 25 min with a sonication power output set at 3. Sonication (Misonix, Farmingdale, NY, USA) was used to increase the efficiency of protein extraction. The crude protein was collected for the next step of purification after the lysate was centrifuged at 14,000× *g* for 20 min.

### 2.3. Purification of ACE2-coreSA Fusion Protein

The ACE2-coreSA fusion protein was extracted from the crude fractions using immobilized metal affinity chromatography. Initially, a 1:1 mixture of the crude protein and 10 mM imidazole (Acros Organics, New Jersey, NJ, USA) was made. The combination was applied to HisPur^TM^ cobalt resin (Thermo Fisher Scientific), and incubated for 1 h under gentle shaking conditions at 4 °C. After loading the mixture onto the column, the flowthrough from the column was collected. The resin was washed at least five times with 10 mM imidazole. To elute the ACE2-coreSA fusion protein, 250 mM imidazole elution buffer was used. A protein concentrator (PES, MWCO = 50 K, Thermo Fisher Scientific) was used to concentrate the elution fractions. For the control experiments, ACE2 protein and coreSA protein with its own gene encoded in the pET-30a(+) vector were expressed, respectively, extracted and purified according to the aforementioned procedures.

### 2.4. Characterization of ACE2-coreSA Fusion Protein

Laemmli 2× sample buffer (Bio-Rad) supplemented with 4% 2-mercaptoethanol (Sigma-Aldrich) was mixed with each sample (crude protein and ACE2-coreSA fusion protein elution) and heated to 95 °C for 5 min. SDS-PAGE was performed using polyacrylamide gels 12% (Bio-Rad), 1× Tris/SDS/glycine buffer (Bio-Rad) for 45 min at 200 V. Next, rapid Coomassie staining solution (Research Products International, Mount Prospect, IL, USA) was used to stain the gels, and 10% methanol and 10% acetic acid buffer were used for overnight de-staining. When SDS-PAGE was completed, a Western blot analysis was conducted. The gels were electrophoretically transferred onto a nitrocellulose membrane (0.45 µm, Bio-Rad) using Trans-Blot semi-dry system (Bio-Rad) for 1 h at 20 V. The membranes were gently rotated in a blocking buffer for 1 h at room temperature (5% bovine serum albumin (BSA, Thermo Fisher Scientific) in TBST [Tris-buffered saline (Bio-Rad) with 0.1% Tween-20 (Bio-Rad)]. Then, three times washes with TBST and overnight incubation at 4 °C with two different primary antibodies (1:1000 dilution): ACE2 monoclonal antibody (mAb) (Thermo Fisher Scientific) and streptavidin mAb (Thermo Fisher Scientific). Next, the membranes were washed three times with TBST and incubated for 1 h in a secondary antibody solution [horseradish peroxide (HRP)-conjugated mouse IgG secondary antibody (R&D Systems, Minneapolis, MN, USA), 1:1000 dilution] at room temperature. Finally, Azure 280 imager (Azure Biosystems, Dublin, CA, USA) was used to image the membranes in the presence of enhanced chemiluminescence (ECL) substrate (Thermo Fisher Scientific).

### 2.5. BCA Protein Assay

Elution protein was quantified by using a BCA protein assay kit (Thermo Fisher Scientific). The BCA assay was carried out by the manufacturer’s instruction methodology. In brief, clean vials were filled with diluted BCA stock (2000 g/mL) to prepare BCA standard dilutions (1500, 1000, 750, 500, 250, 125, and 25 µg/mL). The working reagent (WR) was prepared by mixing BCA reagents A and B (50 parts and 1 part, respectively). In addition, 200 µL of WR and 30 µL of elution protein were combined with each standard. In the final step, a SpectraMax absorbance microplate reader (Molecular Devices, San Jose, CA) was used to measure the absorbance at 562 nm after a 30-min incubation period at 37 °C. The standard curve was built using the reading of each standard versus concentration, and the amount of eluted protein was estimated using the calibrated equation. Experiments were completed in triplicate and reported as mean ± standard deviation.

### 2.6. Spike Protein (S) Pseudotyped Lentivirus Production and Characterization

#### 2.6.1. Plasmid/PEI Complex Preparation

Polyethylenimine (PEI) (Sigma-Aldrich) was prepared at a concentration of 1 mg/mL and sterilized by a 0.22-µm pore size filter (Milliporesigma, Burlington, MA, USA). Then, 6 µg of each plasmid: pCMV-dR 8.2 packaging plasmid for a virus structure that was expressing HIV proteins (Tat, Gag-Pol, and Rev) (Addgene), pSFFV-GFP the tracker EGFP-encoding plasmid (Addgene), and pCMV14-3X-Flag-SARS-CoV-2-S plasmid that expressing SARS-CoV-2 wild type spike protein (Addgene) were mixed with PEI solution at N/P (PEI: DNA) ratio of 5:1. The complex was incubated at room temperature for 30 min before adding them to human embryonic kidney cell line (HEK293T, ATCC, Manassas, VA, USA).

#### 2.6.2. Production and Concentration of S-Pseudotyped Lentivirus

HEK293T cells were seeded with equal density (1.7 × 10^5^ cells/cm^2^) in three T-75 flasks and maintained in Dulbecco’s modified Eagle’s medium (DMEM) (Thermo Fisher Scientific) with 10% fetal bovine serum (FBS) (Thermo Fisher Scientific), and 1% penicillin-streptomycin (Sigma-Aldrich). All the flasks were transfected with the prepared PEI/DNA complex when the confluency of cells reached 80% confluency. Virus-containing media were collected every 24 h for 3 days, and fresh culture media were added to the cells. Virus-containing supernatant was collected and put through a 0.45 µm syringe filter (Thermo Fisher Scientific). For virus spinning down, HEK293T culture supernatants containing virus particles were concentrated using protamine sulfate (Sigma-Aldrich). Briefly, the supernatant was mixed with an equal volume of protamine sulfate (320 µg) and incubated at 37 °C for 1 h. The mixture was then centrifuged for 20 min at 4 °C at 10,000× *g*. In 1 mL phosphate-buffered saline (PBS) (Thermo Fisher Scientific), the viral pellet was resuspended and stored at −80 °C.

#### 2.6.3. S-Pseudotyped Lentivirus Characterization

A dot blot assay was performed by separately adding 2 µL of the wild-type *S*-pseudotyped and native lentivirus to a nitrocellulose membrane. The membrane was blocked for 1 h at room temperature using a blocking buffer (5% *w*/*v* BSA in TBST). Then, the membrane was incubated in the anti-FLAG M2 mAb (Sigma-Aldrich) and SARS-CoV-2 S1 mAb (Thermo Fisher Scientific) for 1 h at room temperature, followed by three times washes with TBST. Next, the membrane was mixed respectively with HRP-conjugated mouse IgG secondary antibody to detect FLAG M2 primary antibody and HRP-conjugated rabbit IgG secondary antibody (Santa Cruz Biotechnology) to detect SARS-CoV-2-S1 primary antibody for 1 h at room temperature. Dynamic light scattering and zeta potential analysis (Brookhaven Instruments Corporation, Holtsville, NY, USA) of *S*-pseudotyped and native lentivirus were conducted to determine the median size and charge of different types of viruses.

### 2.7. Establishing and Characterization of ACE2-Expressing HEK293T Cells

HEK293T cells were seeded at 1.7 × 10^5^ cells/cm^2^ within 10 mL of DMEM with 10% FBS and 1% penicillin-streptomycin in a T-75 flask. The next day, 5 µL of pcDNA3.1-hACE2 plasmid (Addgene) was mixed with 0.1 mL of PEI for 30 min and then added to the cells for transfection. The culture medium was replaced with a fresh one. After 48 h, 500 µg/mL G418 disulfate (Sigma-Aldrich) was used for selection for up to four weeks. The stable HEK293T cell line expressing hACE2 (HEK293T-ACE2) on the surface of the outer cell membrane was confirmed by incubating HEK293T-ACE2 cells with anti-ACE2 mAb for 1 h at room temperature. Following three gentle PBS washes, cells were treated for 25 min at room temperature with a fluorescent secondary antibody: anti-mouse IgGκ binding protein conjugated with red fluorescent dye CruzFluor^TM^ 594 (Santa Cruz Biotechnology). A Leica DMi8 microscope equipped with a Leica EC3 camera (Leica Microsystems, Wetzlar, Germany) was used to image the cells. Western blot analysis was performed in HEK293T-ACE2 cells by using RIPA lysis buffer (Thermo Fisher Scientific). Briefly, after performing SDS-PAGE for the cell lysate, the gel was transferred onto the nitrocellulose membrane. ACE2 mAb and HRP-conjugated mouse IgG secondary antibodies were used for Western blot detection.

### 2.8. Neutralization Assays

We mixed 200 µL of biotin-coated polystyrene particles with green fluorescent (diameter = 0.15 µm) and red fluorescent (diameter = 0.56, 0.8, 2, 5 µm) colors (0.1% *w*/*v*) (Spherotech, Lake Forest, IL, USA), respectively, in 0.1 mL of ACE2-coreSA purified protein solution (100 µg/mL) and gently shook the mixture for 30 min at 4 °C to allow for biotin-streptavidin binding. Particles were collected by centrifugation at 6000× *g* for 5 min, followed by three times washes with 1× PBS to remove unbound ACE2-coreSA proteins. Next, *S*-pseudotyped lentivirus (4 × 10^5^ TU/mL) was mixed with ACE2-coreSA coated particles for 1 h to allow linkage between ACE2 and spike protein. Then, particles were collected by centrifugation at 6000× *g* for 5 min. HEK293T-ACE2 cells were seeded at 5 × 10^4^ cells/cm^2^ on 6-well plates with DMEM supplemented with 10% FBS and 1% penicillin/streptomycin. Then, cells were treated with coated and bare fluorescent polystyrene particles of various sizes 0.15, 0.56, 0.8, 2, and 5 µm. Five hours post-treatment, the conditioned medium was replaced with fresh medium after cells were gently washed three times with 1× PBS. The Leica DMi8 microscope and SpectraMax absorbance microplate reader were used to evaluate the treatment efficacy in blocking virus infection. Additionally, the neutralization assay was conducted by using 0.1 mL of soluble ACE2-coreSA and ACE2 (both with 100 µg/mL) to mix with 1 mL of *S*-pseudotyped lentivirus for 1 h to allow spike protein-ACE2 binding. ACE2 protein and coreSA protein were used separately as positive controls.

### 2.9. Endocytosis of Particles by HEK293T-ACE2 Cells

HEK293T-ACE2 cells were treated separately with bare particles (i.e., biotin-coated fluorescent polystyrene particles with sizes ranging from 0.15 to 5 µm) for 24 h. The particles, not taken up by the cells, were gently washed away twice with 1× PBS. Then, the cells were treated with 0.02 µg/mL trypan blue to bleach any fluorescence in the extracellular milieu. The degree of endocytosis was determined by the fluorescent images taken by a Leica DMi8 microscope equipped with a Leica EC3 camera.

### 2.10. Cell-Based Antiviral Assay

A cell-based antiviral assay with spike protein pseudotyped lentivirus was performed to determine the half-maximal effective concentration of ACE2-coreSA or ACE2. HEK293T-ACE2 cells were infected with the virus in the presence of increasing concentrations of soluble ACE2 and ACE2-coreSA, respectively. After 48 h of treatment, the fluorescence of GFP expressed in cells (indicating the level of viral infection) was monitored and quantified by the SpectraMax microplate reader. The percentage of inhibition was calculated by subtracting the background (number of fluorescent in untreated/infected cells) and normalizing it to uninfected cells (without virus). GraphPad Prism software was used to perform non-linear regression analysis and interpolate EC_50_ values.

### 2.11. Statistical Analysis

The data were all reported as mean ± standard deviation and came from three separate experiments. GraphPad Prism software version 8.0 was used to analyze all data via a one-way analysis of variance (ANOVA) test followed by the Tukey test. Statistical significance was defined as a *p*-value < 0.05.

## 3. Results and Discussion

### 3.1. ACE2-coreSA Fusion Protein Expression, Purification, and Characterization

**Figure 2:**
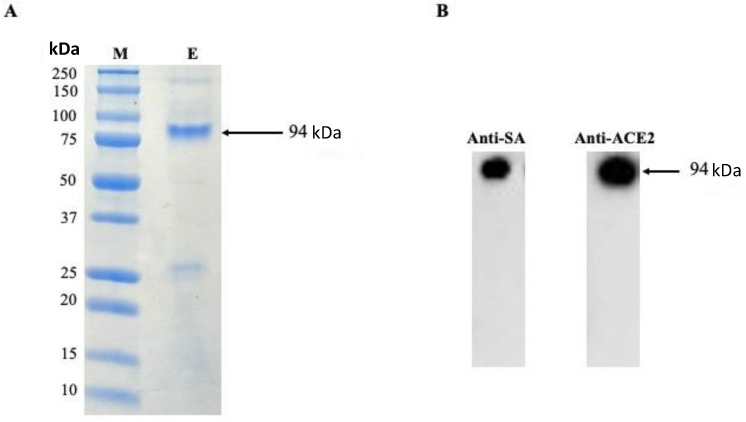
(**A**) Purification of human ACE2-coreSA protein using HisPur^TM^ cobalt resin, (M) All blue protein standard, (E) purified human ACE2-coreSA protein; (**B**) Western blot of purified human ACE2-coreSA protein with anti-streptavidin mAb and anti-ACE2 mAb, respectively. The estimated fusion protein size is 94 kDa. Uncropped blots and the intensity ratio of coreSA and ACE2 Western blot bands were given in Figure S2.

DNA gel electrophoresis was used to validate the gene sequences for ACE2 and coreSA from pET-30a(+) cloned by PCR. As shown in Figure 1B, on an agarose gel, the ACE2 PCR product had a size of around 2.1 kb, while the coreSA PCR product had a size of about 0.4 kb. According to these PCR results, both gene sequences were successfully cloned into pET-30a(+). The coreSA sequence is 387 bp (about 14 kDa), and the ACE2 sequence is 2178 bp (about 80 kDa). Hence, the fusion protein’s estimated molecular weight is roughly 94 kDa. Cell lysates subjected to SDS-PAGE examination revealed a protein band with an MW of 94 kDa (Figure 2A). It is noteworthy that the coreSA forms a tetramer at 37 °C. Therefore, it leads to approximately 400 kDa coreSA-ACE2 fusion protein on tethered biotin-coated particles being used for this study. The gel images (shown in Figure 2) revealed only one band at 94 kD (i.e., monomer of ACE2-coreSA fusion protein) because the fusion protein exhibited monomer configuration under the denatured condition (heated to 95 °C for 5 min). When ACE2-coreSA fusion protein was incubated at 60 °C, four bands were detected. As shown in Figure S1, the upper band is the smear of tetramer and trimer, the middle one is the dimer, and the lower one is the monomer. ACE2-coreSA fusion protein concentration detected by the BCA assay was 269.8 μg/mL. Western blot analysis further confirmed the purified ACE2-coreSA protein by anti-ACE2 mAb and anti-SA mAb (Figure 2B).

### 3.2. Production and Characterization of S-Pseudotyped Lentivirus

Instead of conducting the SARS-CoV-2 virus study in a biosafety level 3 lab, SARS-CoV-2 wild-type spike protein (S), pseudotyped lentivirus, was produced for a biosafety level 2 operation. By using cationic PEI, HEK293T cells were co-transfected with pCMV-dR8.2 packaging plasmid (Addgene) for virus structure that expresses HIV proteins (Tat, Gag-Pol, and Rev), pSFFV-GFP plasmid (Addgene) for EGFP expression, and pCMV14-3X-Flag SARS-CoV-2-S plasmid (Addgene) for expressing the spike protein (i.e., from SARS-CoV-2 wild-type). Then, the produced S-pseudotyped lentivirus was used to infect ACE2-expressing HEK293T (HEK293T-ACE2). Dot blot analysis and dynamic light scattering were performed to verify the spike protein expression on the outer membrane of the lentivirus.

**Figure 3:**
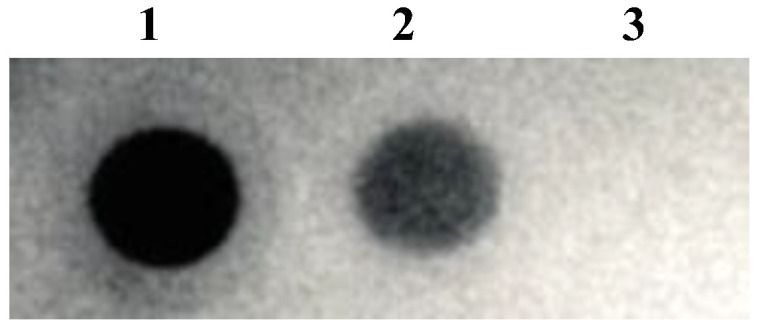
Dot blot analysis using (**1**) anti-FLAG M2 mAb, (**2**) anti-SARS-CoV-2-S1 mAb, and (**3**) native lentivirus was used as a negative control.

The existence of spike protein on the surface of the virus was confirmed through dot blot analysis using anti-FLAG M2 mAb (Sigma-Aldrich) and anti-SARS-CoV-2-S1 mAb (Thermo Fisher Scientific) (Figure 3(1) and (2)). The negative control was the native lentivirus without spike protein (Figure 3(3)). *S*-pseudotyped lentivirus size was determined by dynamic light, as shown in Table 1. The *S*-pseudotyped virus median size was ~21 nm larger than the native lentivirus. The one without spike protein and spike protein median diameter is about 10.6 nm which is comparable to the median diameter of the spike virus (10 nm) reported [32]. The zeta potentials were determined to be negative in both types of viral nanoparticles.

### 3.3. ACE2-Tethered Polystyrene Particles Block the Entry of S-Pseudotyped Lentivirus

**Figure 4:**
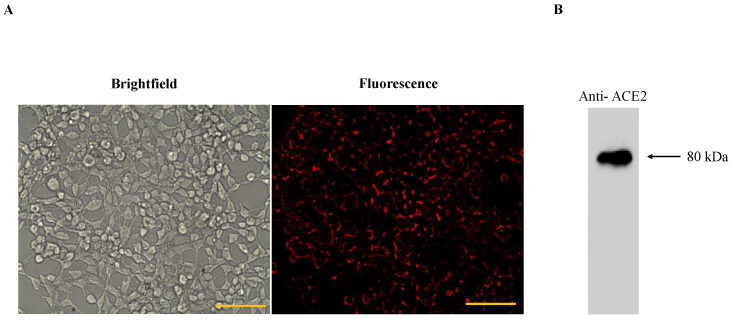
(**A**) Brightfield and fluorescence images of HEK293T-ACE2; anti-mouse IgGk binding protein conjugated with red fluorescent dye CruzFluor™ 594 confirmed the expression of ACE2 receptor on HEK293T cells. Scale bar denotes 100 μm; (**B**) Western blot of HEK293T-ACE2 using anti-ACE2 mAb and HRP-conjugated mouse IgG secondary antibody. The uncropped blot and the intensity ratio of the ACE2 Western blot band are given in Figure S3.

The outer cell membranes of HEK293T-ACE2 cells have a stable expression of ACE2 receptors which is confirmed by immunofluorescence detection (Figure 4). HEK293T-ACE2 cells were treated with different sizes of ACE2-tethered fluorescent polystyrene particles (0.15, 0.56, 0.8, 2, and 5 µm). The physical properties of particles, such as size, shape, and surface, are important parameters for cellular uptake. The focus of this study is on the size impact of ACE2-tethered particles on the enhancement of virus-cell binding inhibition (i.e., blocking viral entry). It has been reported that gold nanoparticles with a size larger than the size of the vesicular stomatitis virus (~50 nm) inhibited the viral binding to cells more efficiently than smaller particles [33]. Therefore, the attention for this work was paid to particles with sizes (i.e., 0.15, 0.56, 0.8, 2, and 5 µm) larger than the size of *S*-pseudotyped viruses (median size ~121 nm given in Table 1).

**Figure 5:**
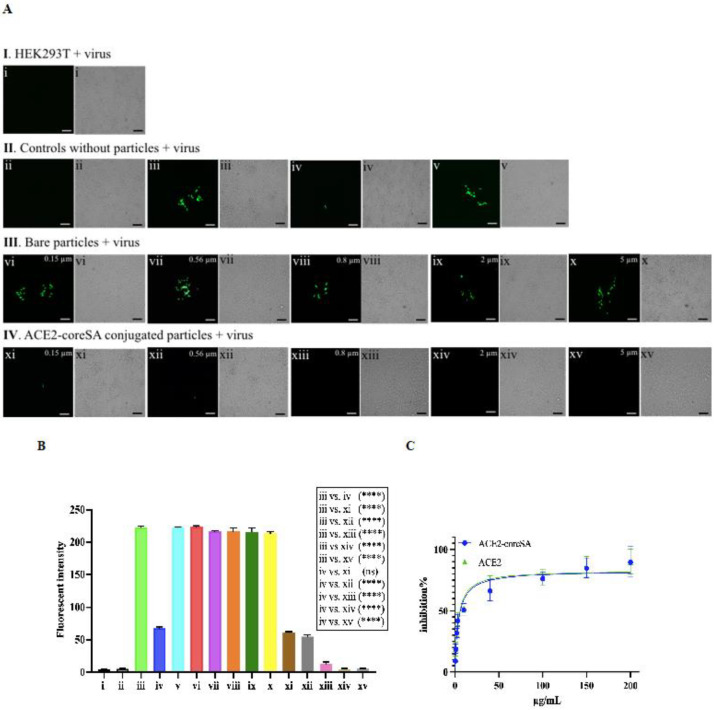
(**A**) Fluorescence and bright-field images of HEK293T and HEK293T-ACE2 cells. (𝐈) HEK293T cells treated with *S*-pseudotyped lentivirus—(i) HEK293T cells treated with virus; (𝐈𝐈) HEK293T-ACE2 cells control samples—(ii) no virus added (negative control), (iii) treated with virus (positive control), (iv) co-treated with virus and soluble ACE2-coreSA, (v) co-treated with virus and soluble coreSA protein only; (**𝐈𝐈𝐈**) HEK293T-ACE2 cells treated with bare polystyrene particles (non-conjugated) and virus—(vi) treated with bare 0.15-µm polystyrene particles and virus, (vii) treated with bare 0.56-µm polystyrene particles and virus, (viii) treated with bare 0.8-µm polystyrene particles and virus, (ix) treated with bare 2-µm polystyrene particles and virus, (x) treated with bare 5-µm polystyrene particles and virus; (**IV**) HEK293T-ACE2 cells treated with ACE2-tethered polystyrene particles and virus—(xi) treated with ACE2-functionalized 0.15-µm polystyrene particles and virus, (xii) treated with ACE2-functionalized 0.56-µm polystyrene particles and virus, (xiii) treated with ACE2-functionalized 0.8-µm polystyrene particles and virus, (xiv) treated with ACE2-functionalized 2-µm polystyrene particles and virus, (xv) treated with ACE2-functionalized 5-µm polystyrene particles and virus. Scale bar = 100 µm; (**B**) Relative fluorescence intensities were quantified by a microplate reader. Statistical analysis was performed using one-way ANOVA followed by Tukey post hoc multiple comparisons tests. Data were presented as mean ± standard deviation (n = 3); ns denotes not significant and (****) denotes *p* < 0.0001; (**C**) The half-maximal inhibitory concentrations of ACE2 and ACE2-coreSA required to inhibit 50% of *S*-pseudotyped lentivirus infection were determined using non-linear regression, respectively.

Our results showed that HEK293T-ACE2 cells treated with ACE2-tethered micro-sized particles (2 and 5 µm) successfully blocked the virus entry to the cells. Intracellular GFP expression levels decreased in a dose-dependent manner with increasing doses of soluble ACE2-coreSA fusion protein, as shown in Figure 5A(II-iv),C. ACE2-coreSA fusion protein’s half-maximal effective concentration (EC_50_) was determined to be ∼10 µg/mL, which is in the same order of magnitude as the previously reported EC value (~23.8 µg/mL) of ACE2-Fc soluble protein for the neutralization of SARS-CoV-2 S pseudoviruses [34]. Likewise, the EC_50_ of ACE2 protein was calculated to be ~10 µg/mL, indicating the functionality of ACE2-SA fusion protein is pretty much the same as ACE2 protein. The parental HEK293T cells (no ACE2 expression on the outer cell membrane) treated with *S*-pseudotyped lentivirus (shown in Figure 5A(I-i)) and HEK293T-ACE2 cells without virus treatment (shown in Figure 5A(II-ii)) have been used as negative controls. As expected, there was no green fluorescence detected under fluorescent microscopy, which was confirmed by fluorescence intensities quantified by a microplate reader (Figure 5B(i,ii)). HEK293T-ACE2 cells were treated with *S*-pseudotyped virus only as a positive control and co-treated with soluble ACE2 and *S*-pseudotyped virus, respectively. The level of green fluorescence detected was less than the GFP expression level of the infected cells using *S*-pseudotyped lentivirus only, which indicates the important role of ACE2 in *S*-pseudotyped virus neutralization (Figure 5A(II-iii,II-iv)). This is also verified by the fluorescence intensities quantified by a microplate reader, as shown in Figure 5B(iii,iv). Another control experiment was conducted using core-SA protein only (i.e., no ACE2 protein). The biotinylated polystyrene particles were decorated with coreSA protein only to further confirm the importance of ACE2 protein for the blocking of viral infection. As shown in Figure 5A(II-v), particles functionalized with coreSA protein only were not able to attach to *S*-pseudotyped lentivirus due to the lack of ACE2-spike protein binding, thereby resulting in similar GFP expression levels illustrated in Figure 5A(II-iii).

In Figure 5A(III-vi,vii,viii,ix,x), HEK293T-ACE2 cells were treated with different bare particles sizes (i.e., unbound with ACE2) in the presence of *S*-pseudotyped lentivirus to confirm the importance of ACE2 protein block the viral infection. *S*-pseudotyped lentivirus particles were not able to attach to the bare particles because when non-ACE2-functionalized particles mixed with *S*-pseudotyped virions cannot interfere with the infection of HEK293T-ACE2 cells via the interaction of spike protein pseudotyped on lentiviruses, thereby leading to a similar level of GFP expression shown in Figure 5A-II-iii. The result was also confirmed by the relative fluorescence intensities (a median value ~ 218) detected by the microplate reader (Figure 5B(iii,vi,vii,viii,ix,x)). This confirms the important role of the ACE2-coreSA protein tethered on biotinylated particles to block the viral infection. Contrary to the previously mentioned results of bare particles, when HEK293T-ACE2 cells were treated with 0.15-µm or 0.56-µm nanoparticles functionalized with ACE2 (Figure 5A(IV-xi,xii), the susceptible cells had a similar decreased degree of infection as soluble ACE2 treatment (Figure 5A(II-iv)). Accordingly, the relative fluorescence intensities (Figure 5B(xi,xii)) had a median value of 60 which is a ~72% decrease from the 218 mentioned above.

In Figure 5A(IV-xiii), no level of GFP expression was detected when 0.8-µm particles tethered with ACE2 were used in the treatment, and the relative fluorescence intensity determined by the microplate reader revealed a median value of 19 (Figure 5B(xiii)), which is ~91% decrease from 218 given above. In Figure 5A(IV-xiv,xv), 2 and 5-µm microparticles tethered with ACE2 nearly completely blocked the entry of *S*-pseudotyped lentivirus into HEK293T-ACE2 with no detection of green fluorescence (Figure 5A(IV-xiv,xv)). The relative fluorescence intensities corroborated this finding (Figure 5B(xiv,xv)). ACE2-tethered nanoparticles with sizes 0.15 and 0.56 µm could bind to *S*-pseudotyped viruses and inhibit viral infection to a significant level (about 72% decrease in fluorescent intensity) compared to those that are bare, as shown in (Figure 5A(IV-xi,xii)). This finding is consistent with a previous study indicating the infectivity of D614G *S*-pseudotyped lentivirus was decreased by ~62% when HEK293T-ACE2 cells were treated with the surrogate viruses tethered with gold nanorods (length = 38 nm and diameter = 10 nm) [35].

**Figure 6:**
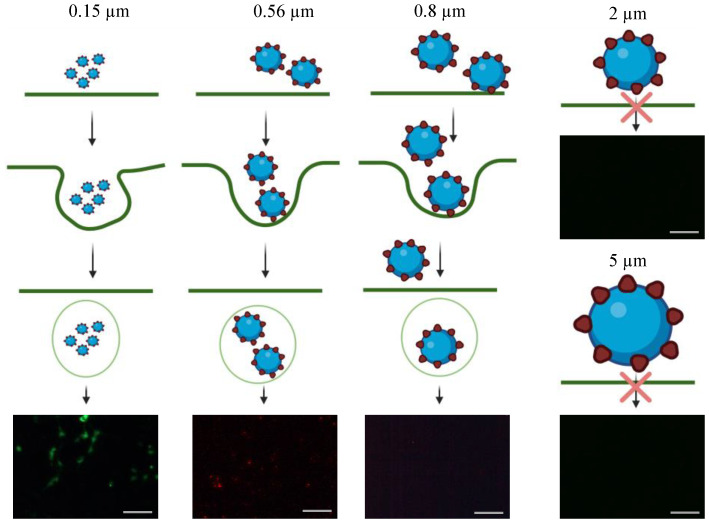
Schematic illustration of HEK293T-ACE2 particles endocytosis. Unlike 0.15 and 0.56 µm fluorescent polystyrene particles, 0.8, 2, and 5 µm fluorescent polystyrene particles were large enough to prevent them from endocytosis by HEK293T-ACE2 cells. The fluorescence images of HEK293T-ACE2 cells were used to illustrate the particles’ endocytosis. The scale bar = 100 µm.

However, in comparison to the antiviral efficacy of using ACE2-tethered 2- and 5-µm microparticles (close to zero fluorescence reading in Figure 5A(IV-xiv,xv), some fractions of HEK293T-ACE2 cells remained infected with *S*-pseudotyped virus treated with ACE2-functionalized 0.15- and 0.56-µm nanoparticles. It is surmised that this observation was caused by endocytosis of virus-nanoparticle complexes (but not with virus-microparticle complexes), thereby resulting in the ensuing infection (as schematically illustrated in Figure 6). Experiments were performed to test this theory by examining the degree of endocytosis of bare particles (i.e., biotin-coated fluorescent particles) with various sizes by HEK293T-ACE2 cells. As shown in Figure 6, the intracellular fluorescence confirmed that HEK293T-ACE2 cells ingested 0.15-µm and 0.56-µm particles and a little of 0.8-µm particles via endocytosis, but the cells were not able to internalize 2- and 5-µm particles.

Our findings show that ACE2-tethered microparticles are more effective decoys than soluble ACE2 because they are more effective at blocking infection, and the treatment with soluble ACE2 requires significantly higher concentrations to achieve comparable levels of inhibition by 2 and 5-µm functionalized particles. Furthermore, the high multivalent impact caused by the high local concentration of ACE2 on functionalized particle surfaces improves the particles’ ability to capture viruses [36]. Antiviral efficacy can be enhanced by the multivalency effect, where the biotinylated particles are used to augment the surface density of targeting ACE2-SA via biotin-streptavidin affinity.

## 4. Conclusions

Good virus inhibitors could be based on preventing the spike protein from binding to the cellular ACE2 receptor [37,38]. Surface functionalization of micro/nanoparticles with target ACE2 molecules can augment their affinity for viral particles, thereby blocking their interaction with the cell membrane and arresting further infection at an early stage. For current and future subsequent coronavirus treatment and prevention, the approach presented in this study has the potential to be developed as a therapeutic product, such as a nasal spray containing ACE2-tethered biocompatible microparticles with sizes ranging from 2–5 microns.

## Figures and Tables

**Table 1:** Dynamic light scattering measurement of size, polydispersity index, and zeta potential of lentivirus and *S*-pseudotyped lentivirus.

	Size (nm)	Polydispersity Index	Zeta Potential (mV)
**Lentivirus**	99.10 ± 12.7	0.251 ± 0.032	−18.25 ± 2.1
***S*** **-pseudotyped lentivirus**	121.25 ± 11.2	0.282 ± 0.030	−23.83 ± 2.0

## Data Availability

The data presented in this study are available on request from the corresponding author.

## Supplementary Materials

The following supporting information can be downloaded at: https://www.mdpi.com/article/10.3390/bioengineering10060652/s1, Figure S1: SDS-PAGE of ACE2-coreSA fusion protein; Figure S2: original Western blots of ACE2-coreSA fusion protein; Figure S3: original Western blot of HEK293T-ACE2.
