# Supplementary material for: Inhibition of SARS-CoV-2 Spike Protein Pseudotyped Virus Infection Using ACE2-Tethered Micro/Nanoparticles
Source: Bioengineering (Basel). 2023 May 26;10(6):652. doi: 10.3390/bioengineering10060652 (PMC10294827; doi:10.3390/bioengineering10060652)
Supplement: Supplementary file 1 [file bioengineering-10-00652-s001.zip › bioengineering-2357386-supplementary.pdf]

Supplemental Materials

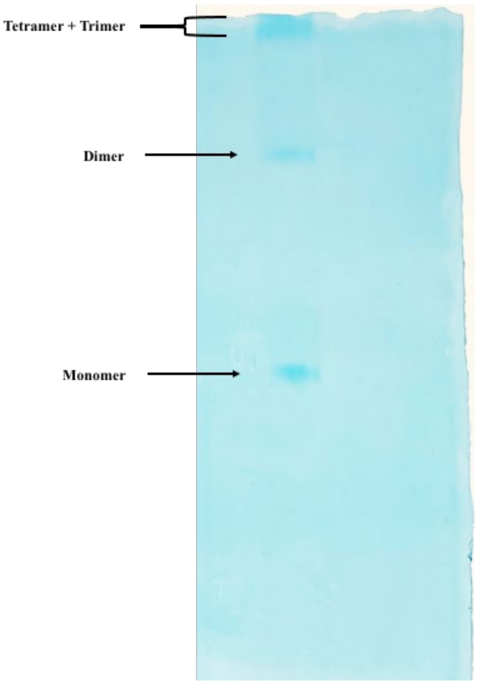

Figure S1. SDS-PAGE gel of ACE2-coreSA fusion protein incubated in the presence of 1% (w/v) SDS and 10% (v/v) mercaptoethanol at 60°C, four bands were detected - upper band is the smear of tetramer and trimer, the middle one is the dimer, and the lower one is the monomer (~94 kDa).

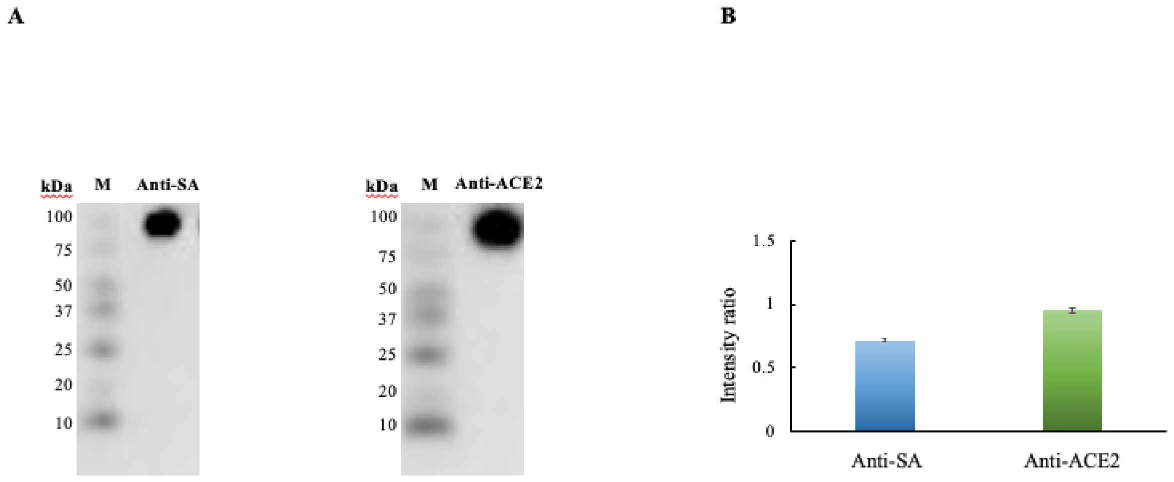

Figure S2. (A) Western blots of purified ACE2-coreSA fusion protein with anti-streptavidin and anti-ACE2 antibodies as primary antibodies, respectively. The estimated fusion protein size ≃ 94 kDa, M = protein marker; (B) The intensity ratio of each band was calculated using NIH ImageJ software.

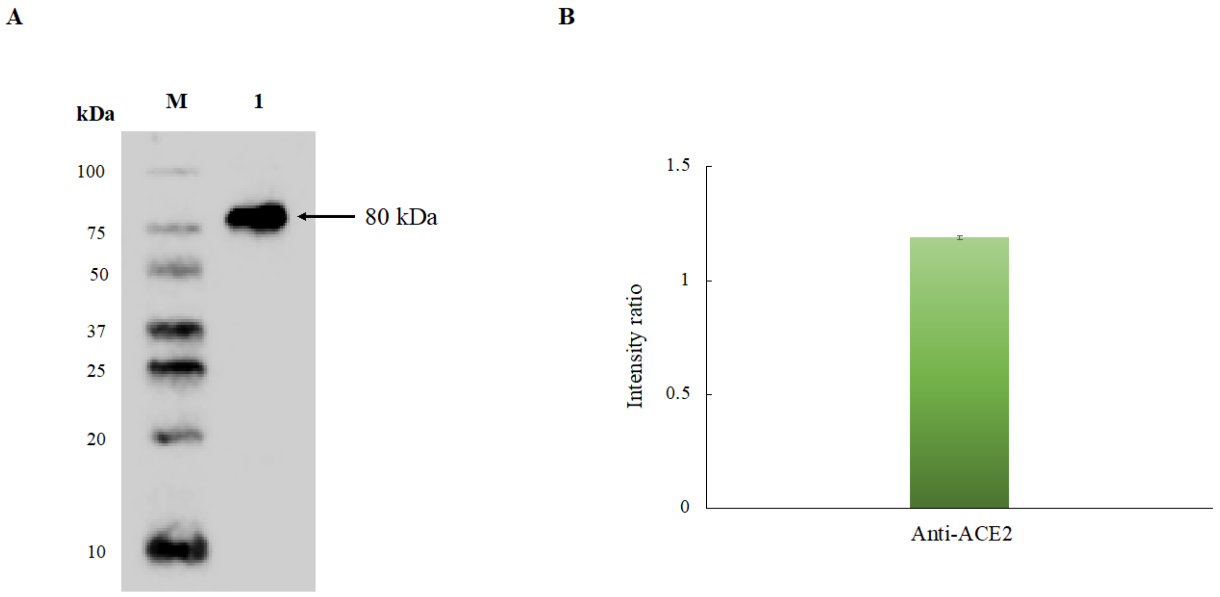

Figure S3. (A) Western blot of HEK293T-ACE2 (lane 1) using anti-ACE2 mAb and HRP-conjugated mouse IgG secondary antibody. M = protein markers; (B) The intensity ratio of ACE2 Western blot band was calculated by NIH ImageJ software.
